# Silica Nanoparticles Disclose a Detailed Neurodegeneration Profile throughout the Life Span of a Model Organism

**DOI:** 10.3390/jox14010008

**Published:** 2024-01-12

**Authors:** Annette Limke, Gereon Poschmann, Kai Stühler, Patrick Petzsch, Thorsten Wachtmeister, Anna von Mikecz

**Affiliations:** 1 IUF–Leibniz Research Institute of Environmental Medicine GmbH, Auf’m Hennekamp 50, 40225 Düsseldorf, Germany; 2 Institute of Molecular Medicine, Proteome Research, Medical Faculty and University Hospital, Heinrich Heine University Düsseldorf, 40225 Düsseldorf, Germany; 3 Molecular Proteomics Laboratory, BMFZ, Heinrich Heine University Düsseldorf, 40225 Düsseldorf, Germany; 4 Biological and Medical Research Center (BMFZ), Medical Faculty, Heinrich-Heine-University, Universitätsstraße 1, 40225 Düsseldorf, Germany

**Keywords:** amyloid disease, *C. elegans*, dopamine, nanomaterial, nervous system, neuropeptides, serotonin

## Abstract

The incidence of age-related neurodegenerative diseases is rising globally. However, the temporal sequence of neurodegeneration throughout adult life is poorly understood. To identify the starting points and schedule of neurodegenerative events, serotonergic and dopaminergic neurons were monitored in the model organism *C. elegans*, which has a life span of 2–3 weeks. Neural morphology was examined from young to old nematodes that were exposed to silica nanoparticles. Young nematodes showed phenotypes such as dendritic beading of serotonergic and dopaminergic neurons that are normally not seen until late life. During aging, neurodegeneration spreads from specifically susceptible ADF and PDE neurons in young *C. elegans* to other more resilient neurons, such as dopaminergic CEP in middle-aged worms. Investigation of neurodegenerative hallmarks and animal behavior revealed a temporal correlation with the acceleration of neuromuscular defects, such as internal hatch in 2-day-old *C. elegans*. Transcriptomics and proteomics of young worms exposed to nano silica showed a change in gene expression concerning the gene ontology groups serotonergic and dopaminergic signaling as well as neuropeptide signaling. Consistent with this, reporter strains for nlp-3, nlp-14 and nlp-21 confirmed premature degeneration of the serotonergic neuron HSN and other neurons in young *C. elegans*. The results identify young nematodes as a vulnerable age group for nano silica-induced neural defects with a significantly reduced health span. Neurodegeneration of specific neurons impairs signaling by classical neurotransmitters as well as neuropeptides and compromises related neuromuscular behaviors in critical phases of life, such as the reproductive phase.

## 1. Introduction

The global burden of neurodegenerative diseases, such as Alzheimer’s (AD) and Parkinson’s (PD) diseases, as well as peripheral neuropathies, is rapidly increasing, with the prevalence of PD more than doubling between 1990 and 2016. In addition to population age, attributing factors include a longer duration of disease and more exposure to risk factors [1]. Among others, one of the great unknowns of neurodegenerative diseases is the critical periods of exposure to risk factors during a life span [2]. Thus, experimental systems that facilitate the observation of specific neurons throughout the entire adult life of an organism and allow for a well-defined exposure to environmental risk factors are needed.

Nano silica has turned out as a reliable tool to induce the degeneration of defined dopaminergic and serotonergic neurons in the animal model *Caenorhabditis elegans* (*C. elegans*). A short life span of 2–3 weeks as an adult hermaphrodite and only 302 neurons that constitute a simple but representative nervous system enables the investigation of the schedule of neurodegeneration in young, middle-aged and old nematodes, e.g., over the entire adult life [3,4]. Neural signaling via dopaminergic, serotonergic or cholinergic neurotransmitters is conserved between *C. elegans* and higher organisms, including mammals [5]. After exposure to the nanoparticles (NPs), fluorescent dopaminergic or serotonergic neurons are observable in transparent reporter worms throughout all life stages, enabling the identification of degenerative phenotypes in specific single neurons [6]. Phenotypes of neurodegeneration include axonal blebbing or beading (axonopathy) and fragmentation and extra branching of dendrites [7]. Degeneration of dendritic integrity impairs the trafficking of cargo, such as neurotransmitters and mitochondria [8,9]. Eventually, these hallmarks of neurodegeneration lead to neural death, and the loss of neurons represents a significant disease pathology [10].

The uptake of silica NPs is facilitated by the fact that *C. elegans* efficiently swallow nanomaterials together with microbial food. Next, nano silica translocates into the gut and the cytoplasm of single intestinal epithelial cells [11,12,13]. Another portal of entry is the reproductive system of the worm. Our previous work indicated that after relocation to nuclei of single vulval cells, nano silica-induced neuromuscular defects of the egg-laying apparatus that is under the control of the hermaphrodite-specific neuron HSN [12,14]. Nano silica promoted a sequence of neurodegenerative events, such as dendritic beading of HSN, impaired serotonergic neurotransmission at the neuromuscular synapse, accelerated failure of vulval muscles and, eventually, intracorporal hatching of progeny from unlaid eggs [14].

In this study, we investigated a panel of specific serotonergic (ADF, HSN, NSM) and dopaminergic neurons (PDE, ADE, CEP) in untreated versus nano silica-exposed fluorescent *C. elegans* reporter worms [15]. ADF, HSN and NSM neurons are integrated into the neural circuits of *C. elegans* that use the neurotransmitter serotonin. ADF is specialized for chemosensory functions and, among others, detects chemicals in the environment. HSN controls a specific motor behavior related to reproduction, e.g., egg-laying and neurodegeneration of HSN can be directly related to this behavioral phenotype. Likewise, a reporter strain expressing green fluorescent protein (GFP) under the control of the dat-1 promoter visualized all dopaminergic neurons PDE, ADE and CEP. To delineate the temporal sequence of neurodegenerative phenotypes, the worms were followed throughout their life span. Four-day-old adult *C. elegans* were subjected to transcriptomic and proteomic analysis to learn more about the gene expression behind neurodegeneration by silica NPs. Based on the results, *nlp-3*, *nlp-14* and *nlp-21* were monitored in young and middle-aged reporter worms, indicating that signaling via neuropeptides may play a role in the nano silica-induced neurodegeneration cascade, e.g., the cross-talk between epithelial cells as portals of NP entry and neuronal cells.

## 2. Results and Discussion

### 2.1. Chronologic Sequence of Nano Silica-Induced Degeneration in Serotonergic and Dopaminergic Neurons

**Figure 1:**
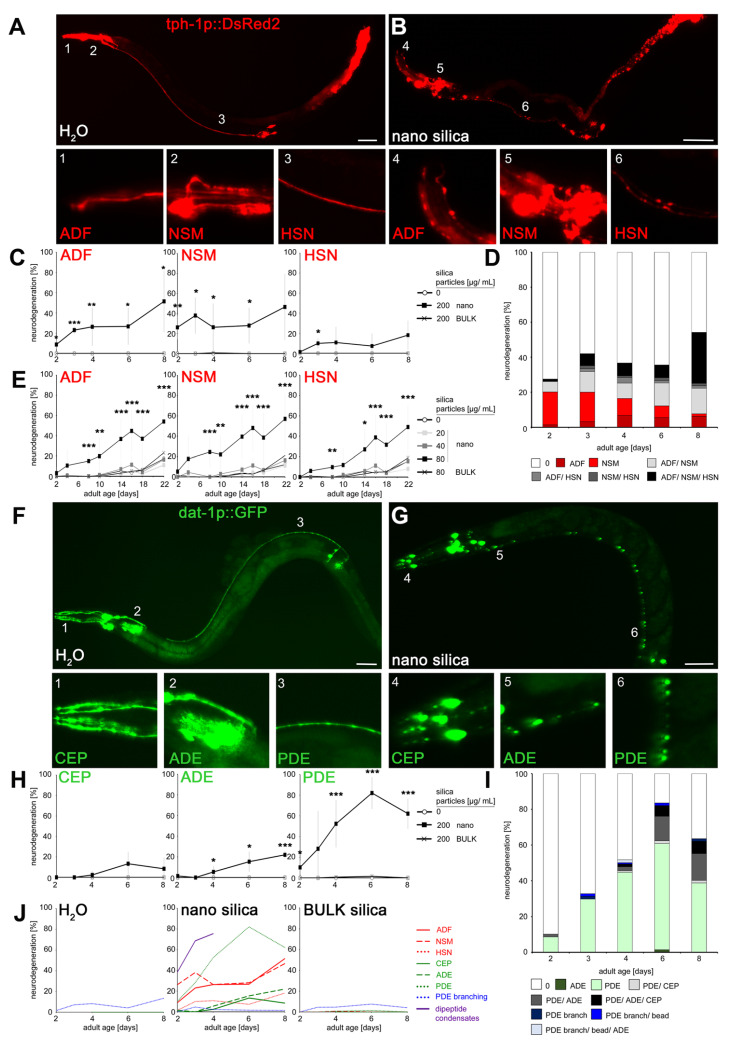
**Patterns of nano silica-induced neurodegeneration during the adult life of the nematode *C. elegans*.** (**A**,**B**) Representative fluorescent micrographs of 8-day-old adult *C. elegans* stably expressing DsRed2 under the control of tryptophan hydroxylase (*tph-1*) in serotonergic neurons. Reporter worms were mock-treated (H_2_O) or exposed to 200 µg/mL nano silica at 20 °C for 7 days. The insets show blow-ups of the ADF dendrites, the NSM processes and the HSN axons (1–6). A discontinuous punctate fluorescence pattern was categorized as neurodegeneration. (**C**) Quantification of neurodegeneration in ADF, NSM and HSN in 2- to 8-day-old worms that were mock-exposed, exposed to 200 µg/mL nano or BULK silica. Line graphs represent means ± SD from 3–6 independent experiments with n = 13–27 per condition per experiment (one-way ANOVA with Tukey’s post hoc test). (**D**) Proportion of neurodegeneration phenotypes in single serotonergic neurons or combinations of ADF, NSM and HSN neurons. (**E**) To determine the lowest observed effect level (LOEL) of neurodegeneration, reporter worms were mock-exposed or exposed to increasing concentrations of nano silica or BULK silica. Values represent means ± SD from 6–8 independent experiments with n = 14–33 nematodes per condition per experiment (one-way ANOVA with Tukey’s post hoc test). (**F**,**G**) Representative, fluorescent micrographs of CEP dendrites, ADE and PDE processes and respective insets (1–6) show 8-day-old reporter worms expressing green fluorescent protein (GFP) under the dopamine transporter (*dat-1*) promoter in dopaminergic neurons. Reporter nematodes were mock-exposed ((**F**), and insets 1–3) or exposed to 200 µg/mL nano silica ((**G**), and insets 4–6) at 20 °C. (**H**) Quantification of neurodegeneration in 2- to 8-day-old worms that were mock-exposed or exposed to 200 µg/mL nano silica or BULK silica. Line graphs show mean values ± SD from 3–6 independent experiments with n = 20–27 per condition per experiment (one-way ANOVA with Tukey’s post hoc test). (**I**) Proportion of neurodegeneration phenotypes in nano silica-exposed worms. Note that PDE branching describes novel outgrowths of neurite processes from the PDE soma. (**J**) Respective line graphs show the time schedule of nano silica-induced neurodegeneration in dopaminergic (green color) or serotonergic (red color) neurons in reporter worms, branching of the dopaminergic PDE neuron (blue color) and the occurrence of dipeptide condensates in intestinal epithelial cells of wild-type worms (N2). Bars, 50 µm. *, *p* < 0.05; **, *p* < 0.01; ***, *p* < 0.001. Note that there is no exclusive neurodegeneration of serotonergic neuron HSN or dopaminergic neuron CEP as they generally co-degenerate with other neurons.

To investigate the time schedule of neurodegeneration in an adult organism, *C. elegans* reporter worms tph-1p::DsRed2 and dat-1p::GFP were observed by fluorescence microscopy throughout the entire life span. In mock-exposed controls, lateral ganglia of ADF neurons localized in the head (Figure 1A; inset 1) and NSM neurons appeared at the anterior bulb of the pharynx (Figure 1A; inset 2). The somata of HSN neurons were located close to the vulva, whereas dendritic processes extended along the ventral nerve cord to the head (Figure 1A; inset 3). After exposition to silica NPs, the fluorescent signal of all dendritic processes changed to dotted patterns (Figure 1B; insets 4–6) that usually indicate dendritic beading and neurodegeneration. Quantification in young and middle-aged *C. elegans* showed that nano silica-induced a significant age-related increase in neurodegeneration in the serotonergic neurons ADF, HSN and NSM compared to mock-exposed controls and reporter nematodes that were exposed to BULK silica particles with a larger diameter (Figure 1C). In contrast to ADF and NSM, neurodegeneration of HSN did not occur exclusively but rather in combination with the other serotonergic neurons (Figure 1D). Neurodegeneration affecting one or more serotonergic neurons increased with adult age in nano silica-exposed worms (Figure 1D, light gray and black bars, 2–8 days). The lowest observed effect level (LOEL) for neurodegeneration was determined at a nano silica concentration of 80 μg/mL (Figure 1E). Notably, the experiment comprised the entire life span (2 to 22 days) and showed that an increase in neurodegeneration in unexposed worms or worms exposed to BULK silica or silica NPs at concentrations of 20 or 40 μg/mL occurred as an intrinsically age-related feature, e.g., in middle-aged and old worms after day 10.

Next, single dopaminergic neurons were observed in young and middle-aged reporter *C. elegans* (dat-1p::GFP; Figure 1F–I). In mock-exposed controls, CEP was located in the head with dendrites extending to the tip of the nose (Figure 1F; inset 1). The neuron ADE is likewise located at the head behind the second bulb of the pharynx (Figure 1F; inset 2), whereas dopaminergic neuron PDE extended next to the dorsal body wall muscles along the lateral side of the body (Figure 1F; inset 3). After exposition to silica NPs, the fluorescent signal of dendritic processes changed to dotted patterns (Figure 1G; insets 4–6). Similar to serotonergic neurons, dendritic beading in dopaminergic neurons CEP, ADE and PDE indicated neurodegeneration by the pollutant. Quantification identified increasing nano silica-induced neurodegeneration in 2- to 8-day-old reporter worms (Figure 1H). Notably, the dopaminergic neurons showed significant differences with respect to sensitivity. The PDE neuron showed a higher sensitivity in comparison to ADE neurons, and CEP neurons were the least sensitive (Figure 1I). The high sensitivity of dendritic beading of PDE also manifested in comparison with the neurodegenerative effects in serotonergic neurons (Figure 1J). Quantification of the neurodegenerative phenotype dendritic branching revealed no differences between unexposed and nano silica-exposed *C. elegans,* suggesting that neuronal beading represents a specific target of nanoparticles (Figure S1A,B). The age-resolved analyses showed that neurodegeneration of PDE dendrites represents an early target of nano silica in 2-day-old nematodes, together with (i) beading of ADF and NSM dendrites, (ii) formation of dipeptide condensates in intestinal cells [13], and (iii) the reproductive defect internal hatch (Figure 1J, Figures S1C,D and S2).

Nano silica-induced neurodegeneration clearly follows a temporal schedule. Over the life span of the organism *C. elegans*, more and different neurons show neurodegeneration, indicating variable vulnerabilities of single dopaminergic or serotonergic neurons. Our previous work is confirmed by identifying a schedule of serotonergic ADF and dopaminergic PDE neurodegeneration, suggesting the vulnerability of young worms and their definition as a susceptible age group with respect to premature reduction of health span by nano silica [16]. As beading precedes the retraction of dendrites to the soma and usually heralds neuronal death, early and specific beading of dopaminergic PDE neurons may serve as a disease model that mimics the death of specific dopaminergic neurons in PD [7,17].

### 2.2. Gene Expression in 4-Day-Old Worms Exposed to Nano Silica

**Figure 2:**
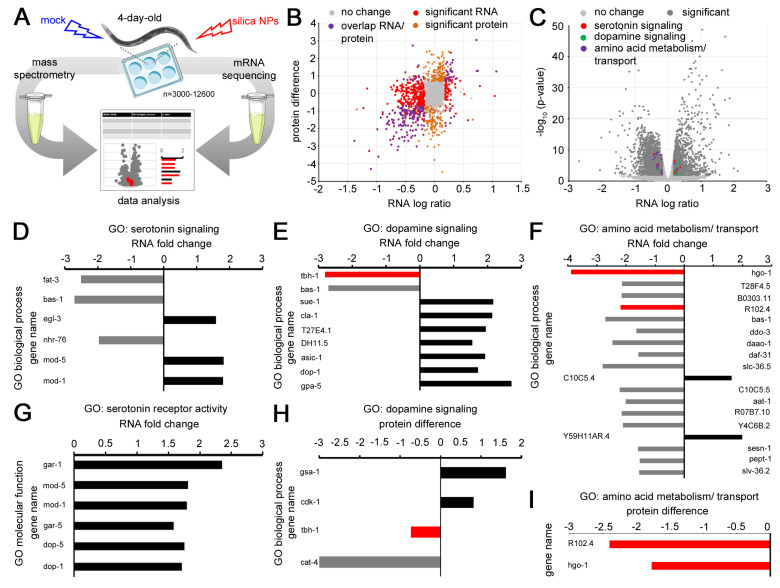
**Gene expression of nano silica-induced neurodegenerative phenotypes.** (**A**) Workflow schematic: Adult wild-type (N2) *C. elegans* were mock-exposed or exposed to 200 µg/mL nano silica for 3 days. For the transcriptomic profiling, worms were lysed, followed by RNA isolation and mRNA sequencing. For the proteomic analyses, total protein was purified and subjected to mass spectrometry. Reads were mapped according to the *C. elegans* reference genome. Genes and gene ontologies (GOs) were categorized using databases of UniProt, PANTHER and WormBase. (**B**) The scatter plot compares differences in gene expression on the protein level (orange) with the mRNA level (red) after exposure to nano silica. Overlap of protein and mRNA levels is displayed in purple. Genes in grey exhibited no significant change in abundance. Each dot represents an individual gene. (**C**) The volcano plot shows significantly enriched or depleted mRNA abundances (*p* < 0.05) in the nano silica-induced transcriptome (dark grey) with involvement in serotonin signaling (red), dopamine signaling (green) or amino acid metabolism and transport (purple). (**D**–**G**) Fold change of genes coding for mRNAs is shown in the gene ontology (GO) categories serotonin signaling (**D**), dopamine signaling (**E**), amino acid metabolism/transport (**F**), and serotonin receptor activity (**G**). (**H**,**I**) Protein difference is shown in the GO category dopamine signaling (**H**) and amino acid metabolism/transport (**I**). Bar graphs display significantly underrepresented (grey), significantly overrepresented (black) and corresponding protein vs. mRNA abundances (red) after nano silica exposure of six independent experiments with n = 3000–12,600 per condition per experiment. MS, mass spectrometry; GO, gene ontology. The protein difference refers to the difference in mean values of log2 transformed normalized intensities.

We showed that silica NPs induced premature degeneration of serotonergic and dopaminergic neurons in young *C. elegans*. Without exposure to the pollutant, neurodegeneration and related neuromuscular defects typically occur later in life, e.g., in middle-aged and old nematodes [12,18]. In order to characterize gene expression in the age cohort that is vulnerable to nano silica exposure, 4-day-old *C. elegans* were analyzed by transcriptomics and proteomics (Figure 2). In comparison with unexposed controls, the gene expression profiles show differential expression of RNA as well as protein in young worms exposed to silica NPs (Figure 2B). Significant changes were observed in candidate genes with a biological role in serotonin signaling, dopamine signaling and amino acid metabolism (Figure 2C). From six genes in the gene ontology (GO) group serotonin signaling, three were less expressed, and three were overexpressed (Figure 2D). In contrast, all candidate genes of the GO group serotonin receptor activity were overexpressed, which might suggest a compensatory role in response to neurodegeneration (Figure 2G). Consistent with this idea, the majority of genes that function in dopamine signaling were overexpressed after nano silica exposure (Figure 2E). However, there was significantly less expression of the gene tbh-1 on RNA and protein levels (Figure 2H). Tbh-1 codes for the protein tyramine β-hydroxylase, which plays a role in the metabolism of the neurotransmitter tyramine, which acts in *C. elegans* behaviors, such as egg laying [19]. Notably, a significant decrease in tbh-1 was likewise identified in *C. elegans* exposed to multi-walled carbon nanotubes [20]. The finding that different nanomaterials target exactly the same gene strongly suggests a role in nano silica-induced neurodegeneration and neural defects. The highest reduction of gene expression was observed for the gene product cat-4, a GTP cyclohydrolase I that has a key function in the biosynthesis of dopamine and serotonin [21]. Two genes were significantly less expressed in the GO group of amino acid metabolism (Figure 2I). While this finding has to be explored further, it fits our previous results showing nano silica-induced impairment of peptide transporter OPT-2/PEP-2 trafficking in the intestinal epithelium [13].

The transcriptomic and proteomic analyses showed that neurodegenerative phenotypes of serotonergic and dopaminergic neurons induced by silica NPs are accompanied by changes in gene expression and protein abundance. In comparison to unexposed controls, young *C. elegans* (4-day-old) exhibited gene profiles with modifications in gene ontology groups that cover neurotransmitters serotonin, dopamine and tyramine. While the exact biological interactions of single candidates have to be determined in more depth, it already becomes evident that nano silica-induced neurodegeneration interacts with neurotransmission and neuronal function. Interference with neural function manifests in neuromuscular defects and can be quantified by observation of behavioral phenotypes.

### 2.3. Silica Nanomaterials Induced the Behavior Defect Internal Hatch

**Figure 3:**
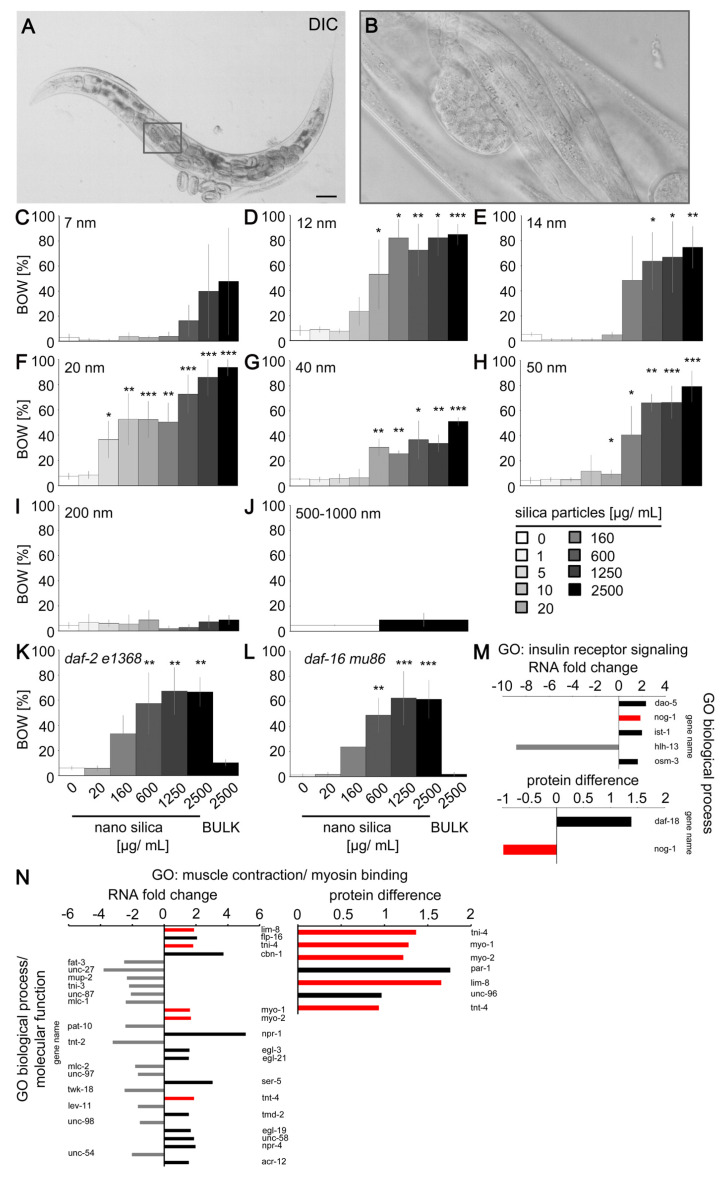
**Nano silica induced the neuromuscular defect internal hatch.** (**A**) Representative differential interference contrast of internally hatched larvae in the parental nematode. (**B**) Inset. Bars, 50 µm. (**C**–**J**) Quantification of the internal hatch defect in 2-day-old wild-type (N2) *C. elegans* that were mock-exposed or exposed to increasing concentrations of silica particles with a diameter of (**C**) 7 nm, (**D**) 12 nm, (**E**) 14 nm, (**F**) 20 nm, (**G**) 40 nm, (**H**) 50 nm, (**I**) 200 nm, (**J**) 500–1000 nm. Values represent means ± SD from 3–6 experiments with n = 26–269, each condition per experiment (two-tailed Student’s *t*-test). Quantification of the internal hatch defect in 2-day-old, long-lived *daf-2* (e1368) mutants (**K**) and short-lived *daf-16* (mu86) mutants (**L**) that were mock-exposed or exposed to increasing concentrations of nano silica (50 nm) or BULK silica (200 nm) for 26 h. Bar graphs represent means ± SD from three experiments with n = 66–200 for each genotype and treatment (one-way ANOVA with Tukey’s post hoc test). (**M**,**N**) Fold change of genes coding for mRNAs is shown in the gene ontology (GO) categories insulin receptor signaling (**M**) and muscle contraction/myosin binding ((**N**), left side), while changes of protein expression are shown in ((**N**), right side). For experimental procedures, see Figure 2A. Bar graphs display significantly underrepresented (grey), significantly overrepresented (black) and overlap of protein/mRNA abundances (red) of six independent experiments with n = 3000–12,600 per condition per experiment. The protein difference refers to the difference in mean values of log2 transformed normalized intensities. *, *p* < 0.05; **, *p* < 0.01; ***, *p* < 0.001; BOW, bag-of-worms; DIC, differential interference contrast; GO, gene ontology group.

Since degeneration of *C. elegans* neurons directly affects corresponding neural circuits and function, we next performed phenotyping of nano silica-induced behaviors. The phenotype internal hatch was chosen for two reasons. The proteomic studies showed an altered protein abundance of tbh-1 that has a role in the metabolism of the neurotransmitter tyramine, which controls egg laying (Figure 2H). Moreover, we demonstrated previously that nano silica-induced beading of neuron HSN corresponded to a reduction of serotonergic neurotransmission and the neuromuscular defect bag of worms (BOW), e.g., internal hatch [14]. In a subpopulation of adult hermaphrodite *C. elegans,* exposure to silica NPs caused malfunction of the vulva muscles, which inhibited egg laying. As a consequence, the progeny hatched within the parental body (Figure 3A,B). To define the physicochemical properties of silica NPs that accelerate the internal hatch phenotype, adult *C. elegans* were exposed to different engineered silica particles (Figure 3C–J). In a concentration-dependent manner, the BOW phenotype was observed in all 2-day-old nematodes exposed to silica particles with a diameter between 7 and 50 nanometers. In contrast, silica particles with larger diameters between 200 and 1000 nanometers (BULK silica) did not significantly induce internal hatch (Figure 3I,J). Thus, we conclude that particle size is the effective property which promotes the behavioral phenotype. Notably, the nanoparticulate silica materials used for the phenotyping experiment possess different properties in addition to diameter size. They appear monodisperse or agglomerate as fractal-like structures and differ with respect to their electrokinetic potential in colloidal suspensions (Figure S3; Table S1). The effective concentrations for induction of BOW varied between 5 μg/mL (20 nm, Aerosil 90 NPs) and 600 μg/mL (14 nm NPs). Three silica nanomaterials had the lowest effect level (LOEL) concentrations of 20 μg/mL.

*C. elegans daf-2* mutants usually live longer than their wild-type counterparts and are more resilient against neurodegeneration [22]. However, silica NPs induced the internal hatch phenotype equally in long-lived *daf-2* mutants and short-lived *daf-16* mutants, suggesting that the insulin signaling pathway has no protective role in nano silica-induced neurodegeneration (Figure 3K,L). Consistent with this, respective transcriptomics and proteomics showed little change in gene expression in the gene ontology group of insulin receptor signaling. The candidate gene nog-1 showed conflicting results, e.g., overrepresentation on the RNA level and lower abundance on the protein level (Figure 3M).

In contrast, the gene ontology group muscle contraction and myosin binding identified changes in the expression of thirty genes (Figure 3N). Five of these genes showed increased abundance on the RNA and protein levels (Figure 3N, red bars). The candidate genes included myo-1, myo-2 and lim-8, which have critical roles in the muscle structure of *C. elegans* as well as invertebrate animal models of neuromuscular disease [23,24,25].

Behavioral phenotyping of young *C. elegans* showed that exposure to silica NPs induced the neuromuscular defect internal hatch during their reproductive phase. Two- and four-day-old worms represent vulnerable cohorts to nano silica since this age group is simultaneously targeted by (i) dendritic beading of neurons ADF, HSN, NSM and PDE, (ii) neurodegeneration, (iii) BOW, and (iv) peptide condensation in the cytoplasm of intestinal cells (this study; [13]). All these defects occur in the time window of the nematode’s reproduction (Figure S4). The induction of internal hatch is nano-specific, as two different BULK silica particles with diameters between 200 and 1000 nanometers were ineffective. In contrast, nano silica used in consumer products interfered with *C. elegans* reproduction at the lowest concentrations. Aerosils 90, 200 and OX 50 that are used in cosmetics, drugs, food, inks, silicone rubber, tires and diverse plastic foils induced internal hatch at LOEL concentrations between 5 and 20 μg/mL. This clearly has ecotoxicological implications due to the growing distribution of nano silica and plastic foils in environmental sinks, such as soils or sediments, which represent natural habitats of wild *C. elegans* and similar nematodes [26,27].

### 2.4. Nano Silica Induced Relocation of Neuropeptide nlp-3 and Axonal Beading in the Command Neuron HSN

**Figure 4:**
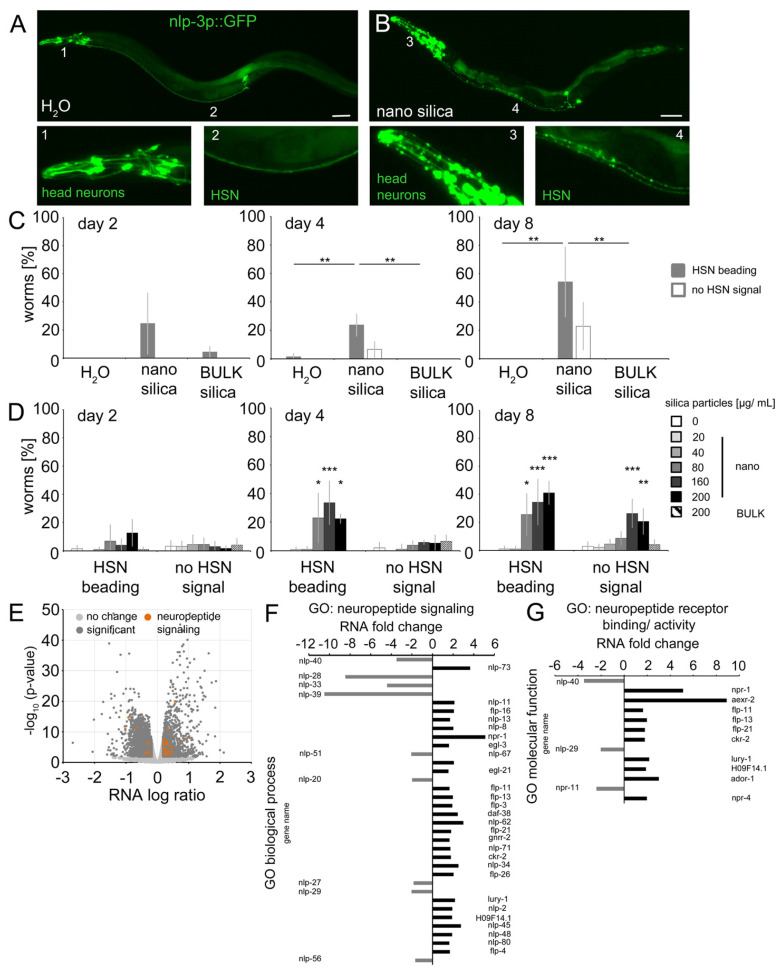
**Nano silica induced relocation of neuropeptide nlp-3 and axonal beading in the command neuron HSN.** Distribution of the neuropeptide nlp-3 was monitored in the HSN axon using reporter nematode nlp-3p::GFP. (**A**,**B**) Representative fluorescent micrographs of 8-day-old adult *C. elegans* were mock-exposed (H_2_O) or exposed to 200 µg/mL nano silica (50 nm diameter) at 20 °C. Bar, 50 µm. A dotted fluorescence pattern was quantified as HSN beading. Loss of fluorescent expression was scored as no HSN signal. (**C**) Phenotypes were quantified in 2- to 8-day-old worms that were mock-exposed or exposed to 200 µg/mL nano silica or BULK silica. Bar graphs depict mean values ± SD of 3–4 independent experiments with n = 20–28 per condition per experiment (one-way ANOVA with Tukey’s post hoc test). (**D**) To determine the lowest observed effect level (LOEL), reporter nematodes were mock-exposed or exposed to increasing concentrations of nano or BULK silica. Values represent mean values ± SD from four independent experiments with n = 20–32 per condition per experiment (one-way ANOVA with Tukey’s post hoc test). (**E**) Volcano plot shows significantly enriched or depleted mRNA abundances (*p* < 0.05) in the nano silica-induced transcriptome (dark grey dots) and genes involved in neuropeptide signaling (orange dots). Fold change of mRNAs is shown in the gene ontology (GO) group neuropeptide signaling (**F**) and neuropeptide receptor binding/activity (**G**). *, *p* < 0.05; **, *p* < 0.01, *** *p* < 0.001; GO, gene ontology; significant, significant change of expression between mock-exposed and nano silica-exposed worms.

**Figure 5:**
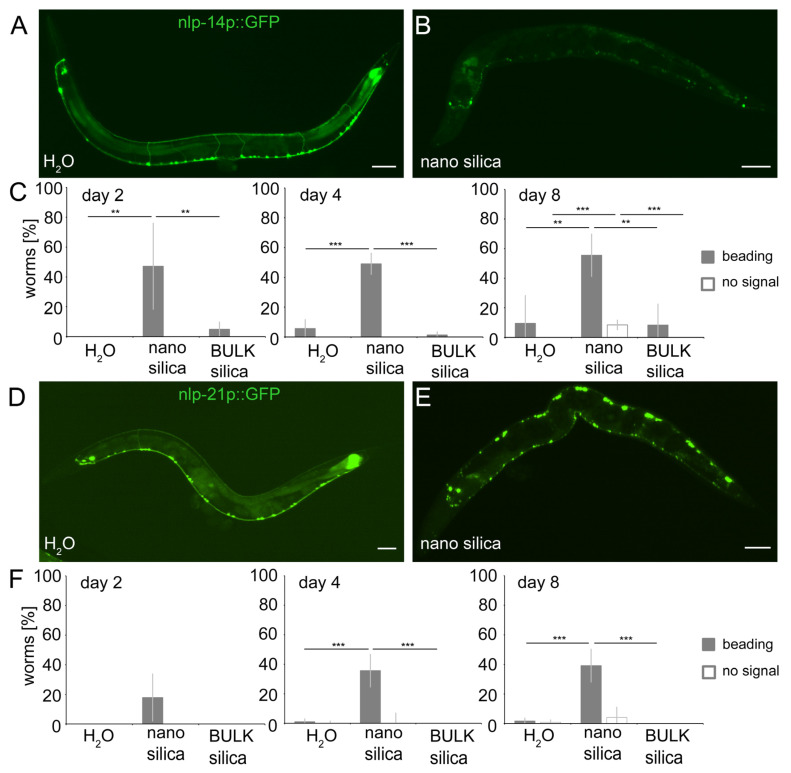
**Nano silica induced relocation of neuropeptides nlp-14 and nlp-21 as well as neural beading.** Localization of neuropeptides in the ventral nerve cord (VNC), dorsal nerve cord (DNC) and commissural trajectories along the dorsoventral axis were examined using (**A**,**B**) nlp-14p::GFP and (**D**,**E**) nlp-21p::GFP. Representative fluorescent micrographs show 8-day-old adult *C. elegans* that were mock-treated (H_2_O; **A**,**D**) or exposed to 200 µg/mL nano silica at 20 °C (**B**,**E**). A discontinuous fluorescence pattern was scored as beading, and loss of fluorescent signal was quantified as no signal. Both phenotypes were categorized as neurodegeneration. (**C**,**F**) Quantification of neurodegenerative phenotypes in 2-, 4- and 8-day-old nematodes that were mock-exposed or exposed to 200 µg/mL nano silica or BULK silica. Bar graphs display mean values ± SD from 3–4 independent experiments with n = 17–29 per condition per experiment (one-way ANOVA with Tukey’s post hoc test). **, *p* < 0.01; ***, *p* < 0.001; BULK, silica particles with a diameter of 500 nm; GFP, green fluorescent protein. Bars, 50 μm.

The proteome of 4-day-old *C. elegans* (N2) exposed to nano silica contained candidates involved in neuropeptide signaling. We, therefore, localized the expression of neuropeptides in respectively exposed reporter worms (Figure 4 and Figure 5 and Figure S5). In mock-exposed 8-day-old *C. elegans,* the neuropeptide-like protein (nlp-3) promoter expressed green fluorescent protein (GFP) in somata and dendrites of head neurons (Figure 4A; inset 1) and the HSN neuron. The fluorescence localized the HSN somata as bright speckles at the midbody close to the vulva (Figure 4A) and HSN dendrites (axons) as continuous signals extending along the ventral nerve cord to the head (Figure 4A; inset 2). After exposure to 200 µg/mL nano silica (50 nm diameter), the fluorescence signals changed significantly (Figure 4B and Figure S5). All dendrites of the head neurons showed a dotted discontinuous fluorescence pattern, indicating beading and neurodegeneration (Figure 4B; inset 3). Likewise, dendrites of the HSN neuron showed a beaded pattern along the ventral side of the body (Figure 4B; inset 4). Quantification of HSN beading in young and middle-aged worms identified a significant increase from day 4 in nano silica-exposed cohorts versus unexposed cohorts and *C. elegans* exposed to BULK silica (Figure 4C). With increased age, a complete loss of the HSN signal was observed, which usually indicates neurodegeneration (Figure S5C). The results were confirmed by concentration range-finding experiments (Figure 4D). Dendritic beading of HSN neurons was identified in 4-day-old and 8-day-old *C. elegans* that were exposed to nano silica concentrations starting from 80 μg/mL. Complete loss of HSN signal was observed in 8-day-old middle-aged *C. elegans* that were exposed to nano silica concentrations starting from 160 μg/mL. Mock exposure or exposure to BULK silica showed no neurodegenerative effects (Figure 4D).

Next, the results from local distributions of nlp-3 expression were compared with gene expression of neuropeptides in 4-day-old *C. elegans* after exposure to nano silica (Figure 4E–G). The volcano plot of RNA levels showed a significant number of genes with a function in neuropeptide signaling in the group of significantly higher abundance (Figure 4E; orange dots). In the GO group neuropeptide signaling, nine genes showed lower RNA levels, and twenty-seven genes showed higher RNA levels (Figure 4F). The maximum was a fivefold increase concerning the RNA level of npr-1. In the GO group neuropeptide receptor binding and activity, three genes showed lower RNA levels, and ten genes showed higher RNA levels (Figure 4G). The maximum was a ninefold increase concerning the RNA level of aexr-2.

The results suggest that silica NPs induced significant changes in neuropeptide expression. With respect to localization, nano silica induced significant redistribution of neuropeptide nlp-3 in head neurons and axons of serotonergic neuron HSN in young and middle-aged worms. This is consistent with the previously reported beading, neurodegeneration and behavioral defects of HSN after nano silica exposure in young worms ([14]; this study). This study adds impaired neuropeptide signaling in the command neuron HSN as a contributor to the nano silica-induced egg-laying defects. Notably, beading and internal hatch were observed simultaneously in young reporter *C. elegans*, showing altered serotonin (tph-1p::DsRed2) and neuropeptide (nlp-3p::GFP) expression (Figures S4–S6). Consistent with this idea, transcriptomics confirmed the role of neuropeptides in nano silica-induced neurodegeneration. The number of genes in the GO groups neuropeptide signaling and neuropeptide receptor activity, as well as an up to ninefold abundance of RNA, identifies this pathway as a biological target of silica NP exposure. Signaling by neuropeptides represents an attractive pathway that may mediate cross-talk between the uptake of silica NPs into intestinal cells, serotonergic neurotransmission of the command neuron HSN and defects of vulval muscle cells, resulting in the behavioral defect internal hatch.

### 2.5. Nano Silica Induced Relocation of Neuropeptides nlp-14 and nlp-21 as Well as Neural Beading

To investigate the effects of silica NPs on neuropeptides with defined expression in *C. elegans* neurons and characterized neural functions, we next subjected gene reporters of nlp-14 (nlp-14p::GFP) and nlp-21 (nlp21p::GFP) to fluorescence microscopy. Mock-exposed nlp-14 and nlp-21 reporters showed expression of the respective neuropeptides in neurons located in the ventral nerve cord (Figure 5A,D) [28]. The somata of the neurons appeared as punctate dots that were connected by thin, continuous lines representing the dendrites. After exposure to nano silica, the fluorescence signal changed to patterns of dendritic beading or a complete loss of dendritic signals, which usually indicates neurodegeneration and complete neural loss (Figure 5B,E). Quantification of young (2- and 4-day-old) and middle-aged (8-day-old) nlp-14 gene reporters identified significant dendritic beading after only one day of exposure with nano silica, whereas mock-exposed or animals exposed to BULK silica showed no neurodegeneration (Figure 5C). In nlp-21 gene reporter worms, phenotypes of neurodegeneration occurred later, e.g., in older age cohorts from day 4 (Figure 5F). Again, mock-exposed and nlp-21 gene reporters exposed to BULK silica showed no neurodegenerative phenotypes.

The results confirm the idea that silica NPs induce neurodegeneration, impairing neurotransmission of serotonergic and dopaminergic neurons and signaling by neuropeptide-like proteins. Since nlp-3 plays a role in pharyngeal pumping, while nlp-14 and nlp-21 signaling modulate locomotion behavior, the versatility of neuropeptide functions may explain the many behavioral defects observed in response to nano silica exposure [12,14,28,29,30]. Specifically, nlp-3 acts together with serotonin in HSN neurons to control egg laying [31], which may explain the sensitivity of vulval muscles and resulting defects in response to exposure to silica nanomaterials (Figure 3).

Nlp-3, nlp-14 and nlp-21 are expressed in neurons and the intestine of *C. elegans* [28,29], which clarifies how silica NPs and other nanoparticulate particles that are mainly taken up by ingestion induce neurodegeneration and impair neural phenotypes. Previous work clearly showed that, in *C. elegans*, long-range neuropeptide signaling from the gut is involved in the cross-talk between intestinal cells and neural cells [32,33,34]. Beyond expression throughout the nervous system, many neuropeptides, including nlp-3, nlp-14 and nlp-21, are expressed in somatic gonads, intestine and vulval hypodermis [29], which all represent entry portals of silica NPs [12].

## 3. Conclusions

**Figure 6:**
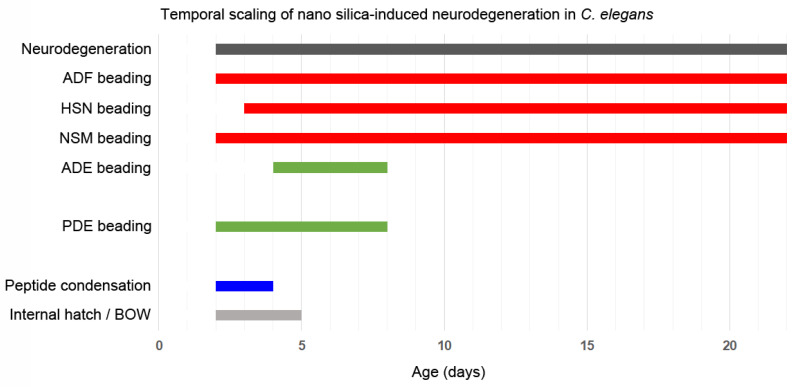
**Schematic shows the chronology of nano silica-induced neurodegeneration throughout the life span of the nematode *Caenorhabditis elegans*.** Neurodegeneration in reporter worms (i) of serotonergic neurons is depicted in red, and (ii) of dopaminergic neurons is shown in green. Notably, unexposed nematodes developed neurodegeneration and axonal beading in old age groups (>10 days).

Neurodegeneration of serotonergic and dopaminergic neurons is an intrinsic feature of aging in the model organism *C. elegans* that can be accelerated by nano silica but not BULK silica. Silica NP-induced neurodegeneration concerns both serotonergic and dopaminergic neurons. Certain neurons, such as serotonergic ADF and dopaminergic PDE neurons, are highly susceptible to the pollutant. Nano silica-induced neurodegeneration followed a time schedule that manifested by simultaneous occurrence of combinations of serotonergic neurons ADF and NSM or in middle-aged *C. elegans* combinations of all serotonergic neurons (ADF, NSM and HSN). The time window of silica NP-induced neurodegeneration coincided with specific behavioral defects. An example represents the internal hatch phenotype that occurred simultaneously with neurodegeneration of serotonergic neurons in 2-day-old young nematodes (Figure 6; Schematic). Notably, internal hatch was observed in silica nanomaterials with different biophysical properties, including those with wide use in consumer products. As *C. elegans* has 56 glial cells in addition to 302 neurons, it will be interesting to investigate the role of astrocytes in neural circuits and related neurobehavior [35] when the nematode is challenged by pollutants, aging or other external and internal exposome factors.

Neurodegeneration and the respective behavioral phenotypes were interrogated by means of transcriptomics and proteomics, which confirmed altered gene expression of serotonergic and dopaminergic neurotransmission pathways. Changes in gene expression were newly identified in the gene ontology group of ‘neuropeptide signaling pathways’, which suggests a role for endocrine signaling as bio-interaction of nano silica. As neuropeptides are expressed and released from intestinal cells and somatic reproductive organs, we have discovered a likely molecular pathway for cross-talk between entry portals of silica NPs and the neural system. Silica NPs enter single intestinal epithelial cells, vulval epithelial cells and the pharyngeal tissue but have not yet been located in neural cells [12]. While neuropeptide signaling has to be investigated in more detail, it already represents an attractive starting point for the comparative data collection on nanoparticulate effects in the model organism *C. elegans* and human cohorts.

The collection of *C. elegans* models for neuronal diseases is expanding and becoming more sophisticated. In this study, we introduce life span resolved analyses of reporters for the serotonergic and dopaminergic nervous systems that are poised to unveil connections among the degeneration of specific neurons, neuromuscular behaviors, age and environmental influences on a broader scale. Investigating genes, neurons and neurobehavior throughout the entire life span of *C. elegans* could ultimately bridge the evolutionary gap between nematode models and human diseases such as AD and PD by enhancing our understanding of the fundamental neurobiology underlying pollutant-induced neurodegeneration. Clinical manifestations of disease, such as locomotion deficits, paralysis, stiffness and even loss of orientation, can be replicated with precision in *C. elegans* and interrogated for respective molecular pathways [36]. Therapeutic interventions, such as the substitution of neurotransmitters dopamine or serotonin, as well as screening for new drugs, can be performed in the invertebrate disease models that provide promising high throughput options.

## 4. Materials and Methods

### 4.1. Particles

Nano silica and BULK silica were purchased as follows. Nano silica with diameters of 7 nm or 14 nm and BULK silica with a diameter of 500–1000 nm were from Sigma-Aldrich (Darmstadt, Germany). Silica NPs with diameters of 12 nm, 20 nm or 40 nm were from Evonik Industries AG (Hanau, Germany). Silica particles with diameters of 50 nm, 200 nm or 500 nm were from Kisker (Steinfurt, Germany). Stock solutions of silica particles were prepared by suspending 25 mg/mL powder in H_2_O. Nano silver (15 nm, NM300K) and silver dispersant control (NM300KDIS) were provided by the European Commission Joint Research Center (JRC, Ispra, Italy). All particle dispersions were serially diluted in H_2_O as indicated. Particles were analyzed by transmission electron microscopy (TEM) and dynamic light scattering (DLS) in H_2_O using the Zetasizer Nano-ZS (Malvern Instruments Ltd., Malvern, UK) as described previously [13,30]. Silica particles with diameters of 500 nm and above were used as BULK control.

### 4.2. Caenorhabditis Elegans Strains

*C. elegans* strains were cultured at 20 °C with live *Escherichia coli* strain OP50 on nematode growth medium (NGM) plates supplemented with yeast extract [37]. All strains were purchased from *Caenorhabditis* Genetics Center (University of Minnesota, Minneapolis, MN, USA): Bristol N2 (wild type), LX975 *vsls97* [*tph-1p::DsRed2* + *lin-15*(+)] [38], BZ555 *egls1* [*dat-1p::GFP*] [39], HA353 *lin-15B&lin-15A(n765)* X; *rtEx247* [*nlp-14p::GFP* + *lin-15*(+)], HA341 *lin-15B&lin-15A(n765)* X; *rtEx235* [*nlp-3p::GFP* + *lin-15*(+)] and HA444 *lin-15B&lin-15A(n765)* X; *rtEx330* [*nlp-21p::GFP* + *lin-15*(+)] [28].

### 4.3. Particle Exposures in Liquid Media

Nematodes were synchronized by isolating eggs with hypochlorite/NaOH and allowed to hatch on NGM plates at 20 °C. As L4 larvae, animals were transferred to liquid medium (S-medium, pH 5.7, with 50 µg/mL carbenicillin and 0.1 µg/mL fungizone) on 96-well plates with 12 mg/mL freshly prepared *E. coli* OP50 [30,40]. For age-resolved experiments, nematodes were supplemented with 5-fluoro-2′-deoxyuridine (FUdR, 1.5 mM final concentration) to maintain synchronization when not stated otherwise. On day 1 of adulthood, worms were mock-treated (H_2_O or dispersant) or exposed to nano silica, nano silver or BULK silica at the indicated concentrations. All experiments were performed at 20 °C.

### 4.4. Microscopy and Quantification of Neurodegeneration

Living reporter nematodes were analyzed from young (day 2) to middle age (day 8) or old age (day 22) on 5% agarose pads with 10 μM NaN_3_ at room temperature. Neurodegeneration was monitored by epifluorescence microscopy with a 60×/1.4 NA Plan Apo objective (Ix70, Olympus, Tokyo, Japan) in 2- to 22-day-old adult hermaphrodite reporter worms. Discontinuous dotted staining pattern along dendrites, processes and axons in the respective neurons was quantified as beading. Outgrowth of new neurite branches from the PDE soma was scored as PDE branching. Loss of fluorescence signal in the HSN axon, the ventral nerve cord (VNC) or dorsal nerve cord (VNC) was scored as no signal. All neural phenotypes were categorized as neurodegeneration. Micrographs were taken with a stereo microscope (SMZ18, Nikon Europe B.V., Amsterdam, The Netherlands) with an SHR Plan Apo 2x objective. The micrograph of the PDE soma was taken with the Ix70 Olympus microscope (see above). The reporter DsRed2 was detected with 561 nm excitation/575–615 nm emission. The reporter green fluorescent protein (GFP) was detected with 488 nm excitation/510–550 nm emission. Image processing was performed with the NIS Elements software (NIKON) (https://www.microscope.healthcare.nikon.com/products/software/nis-elements) or Metamorph image analysis software package (Molecular Devices, Sunnyvale, CA, USA).

### 4.5. Microscopy and Quantification of Peptide Condensates

The assay was performed as previously described [13]. Briefly, worms were incubated in a 1 mM ß-Ala-Lys-AMCA solution in M9. After 2–3 h, animals were washed at least four times in M9 and placed on 5% agar pads with 10 mM NaN_3_. Peptide condensation was quantified by epifluorescence microscopy, and micrographs were taken with a 60×/1.4 NA Plan Apo objective (Ix70, Olympus, Tokyo, Japan) in single intestinal cells of 2- to 4-day-old adult wild-type (N2) nematodes. ß-Ala-Lys-AMCA staining was detected by 358 nm excitation/430 nm emission. Image processing was performed with the Metamorph image analysis software package (Molecular Devices, Sunnyvale, CA, USA) and assembled with Photoshop (Adobe, Redmond, WA, USA).

### 4.6. Internal Hatch

Wild-type (N2) and *daf-2* or *daf-16* mutants were transferred to a liquid medium into 96-well plates without FUdR. On day 1 of adulthood, nematodes were mock-exposed (H_2_O) or exposed to silica particles with different biophysical properties as indicated. After 26 h, worms were mounted on 5% agarose pads, and worms showing internally hatched larvae (bag-of-worms, BOW) were scored as BOW-positive.

### 4.7. C. elegans Sample Preparation for Transcriptomic and Proteomic Analyses

*C. elegans* wild-type (N2) were transferred as L4 larvae in liquid medium in 6-well plates containing 20 mg/mL *E. coli* OP50 and FUdR (1.5 mM final concentration) to maintain age-synchronization. Nematodes were mock-exposed (H_2_O) or exposed to 200 µg/mL nano silica on day 1 of adulthood. After 72 h, half of the worms were prepared for the transcriptomic analysis, and the other half was used for the proteomic analysis. Six independent experiments were prepared with n = 3000–12,600 per condition per experiment.

### 4.8. Worm Lysis, Protein Determination and PAA Gel Quality Control

Nematodes were collected, washed with M9 and spun down. After the last washing step, the supernatant was discarded, and the pelleted worms were frozen in liquid nitrogen. Protein lysates were prepared by bead milling of worms in lysis buffer containing urea, thiourea and 3 [(3 Cholamidopropyl)dimethylammonio] 1 propanesulfonate (CHAPS), as described earlier in detail [41]. Subsequently, the protein concentration of the samples was determined using the Pierce 660 nm protein assay (Thermo Fisher Scientific, Waltham, MA, USA), and quality control was carried out by separating proteins in 4–12% Bis(2-hydroxyethyl)amino-tris(hydroxymethyl)methan polyacrylamide gels (Novex NuPAGE, Thermo Fisher Scientific) with subsequent silver staining.

### 4.9. Protein Sample Preparation for Mass Spectrometric Analysis

Protein samples were prepared for mass spectrometric analysis by in-gel digestion, essentially as described [41]. Briefly, 5 µg of protein per sample was shortly stacked into 4–12% Bis(2-hydroxyethyl)amino-tris(hydroxymethyl)methan polyacrylamide gels (Novex NuPAGE, Thermo Fisher Scientific) and stained with Coomassie brilliant blue. Protein-containing bands were further processed for mass spectrometric analysis by reduction with dithiothreitol, alkylation of cysteine residues with iodoacetamide and digestion with trypsin in ammonium bicarbonate solution. Finally, peptides were extracted from the gel piece and vacuum dried, and 500 ng was prepared in 0.1% (*v*/*v*) trifluoroacetic acid in water for mass spectrometric analysis.

### 4.10. Mass Spectrometric Analysis of the C. elegans Proteome

Prepared peptides were first separated over 2 h by an UltiMate 3000 rapid separation liquid chromatography system (RSCL, Thermo Fisher Scientific) on C18 material, essentially as described earlier [41]. Subsequently, the peptides were sprayed via a nano-source interface into a QExactive plus mass spectrometer (Thermo Fisher Scientific) operated in data-dependent positive mode. Tandem mass spectra were recorded as follows: firstly, survey scans were carried out (resolution 140,000, AGC target 3,000,000, maximum ion time 80 milliseconds, scan range 350 to 2000 *m*/*z*, profile mode), and secondly, fragment spectra were recorded after quadruple isolation (2 *m*/*z* isolation window) of up to top ten 2- and 3-fold charged precursor peptides (resolution 17,500, AGC target 100,000, maximum ion time 60 milliseconds, scan range 200 to 2000 *m*/*z*, centroid mode). Already fragmented precursors were excluded from fragmentation for 100 s.

The analysis of mass spectrometric data was carried out with MaxQuant version 1.6.10.43 (Max Planck Institute for Biochemistry, Planegg, Germany), which was used for peptide and protein identification and quantification with standard parameters, if not stated otherwise. *C. elegans* protein sequences from the UniProt knowledge base (UP000001940, downloaded on 27 September 2019) were used for the searches. The “match between runs” function was enabled, as well as label-free quantification (LFQ).

Quantitative protein data was subsequently analyzed within the Perseus framework (version 1.6.6.0, Max Planck Institute for Biochemistry, Planegg, Germany). Only proteins were considered for analysis identified with at least 2 different peptides and 5 valid quantitative (LFQ) values at least in one group. Before statistical analysis, normalized (LFQ) intensities were log2 transformed, and missing values were filled in with values randomly drawn from a downshifted normal distribution (downshift: 2.2 standard deviations, width 0.3 standard deviations). Differentially abundant proteins were detected using the Student’s *t*-test-based significance analysis of microarrays method [42] using an S^0^ of 0.6 and a false discovery rate of 5%.

### 4.11. RNA Isolation

Worms were collected, washed with M9 and spun down. After the last wash step, the supernatant was discarded, and the pelleted worms were transferred to 2 mL RNase-free Eppendorf tubes containing 1 mL of cooled TRIzol. Samples were vortexed for 30 sec and shaken for 20 min at 1400 rpm at 4 °C. For further lysis, worms were subjected to a swing rocket with a 7 mm steel bead for 5 min at room temperature (RT) and spun down for 10 min at 14,000 g at 4 °C. The supernatant was transferred to a new RNase-free Eppendorf tube and incubated for 5 min at RT to permit complete dissociation of nuclear proteins. An additional 260 µL chloroform was added, incubated for 3 min at RT and spun down for 15 min at 12,000 g at 4 °C. The aqueous phase was transferred into a new RNase-free Eppendorf tube. RNA was precipitated after the addition of 500 µL 2-propanol: the mixture was inverted, incubated for 10 min at RT and spun down for 10 min at 12,000 g at 4 °C. After the removal of the supernatant, the pellet was washed twice with 1 mL 70% ethanol and spun down for 5 min at 7500 g at 4 °C. The supernatant was removed, and the pellet air was dried and resuspended in 50 µL RNase-free H_2_O. To dissolve the RNA, the samples were heated for 10 min at 60 °C. RNA isolation was completed with a DNA-digest kit according to the manufacturer’s instructions (DNA-free^TM^ DNA Removal Kit, Invitrogen^TM^, Thermo Fisher Scientific, MA, USA). RNA samples were stored at −80 °C.

### 4.12. RNA Seq Analyses

DNase digested total RNA samples used for transcriptome analyses were quantified (Qubit RNA HS Assay, Thermo Fisher Scientific, MA, USA), and quality was measured by capillary electrophoresis using the Fragment Analyzer and the ‘Total RNA Standard Sensitivity Assay’ (Agilent Technologies, Inc., Santa Clara, CA, USA). All samples in this study showed high-quality RNA Quality Numbers (RQN; mean = 9.2). The library preparation was performed according to the manufacturer’s protocol using the ‘VAHTS mRNA-Seq V3 library prep kit for Illumina’. Briefly, 200 ng total RNA was used for mRNA capturing, fragmentation, the synthesis of cDNA, adapter ligation and library amplification. Bead-purified libraries were normalized and finally sequenced on the HiSeq 3000/4000 system (Illumina Inc., San Diego, CA, USA) with a read setup of SR 1 × 150 bp. The Illumina bcl2fastq tool (v2.20.0.422) was used to convert the bcl files to fastq files, as well as for adapter trimming and demultiplexing.

### 4.13. Transcriptome and Proteome Analyses

The identified proteins and mRNAs were classified according to their biological process, molecular function and cellular component using the UniProt [43] database. Further, differentially abundant proteins and mRNAs were categorized into serotonin (receptor) signaling, dopamine signaling, neuropeptide signaling, neuropeptide receptor binding/activity, amino acid metabolism/transport, insulin receptor signaling and muscle contraction/myosin binding using the following databases: UniProt [43], PANTHER [44] and WormBase [45].

### 4.14. Statistical Analyses

Neurodegeneration and behavioral data. Data are presented as mean ± SD from at least three independent biological replicates unless stated otherwise. Volcano and scatter plots, bar graphs and line graphs were created in Excel (Microsoft Office, 2019 MSO)). Statistical analyses were performed with the two-tailed Student’s *t*-test (Microsoft Excel) for between-group comparisons, one-way ANOVA with Tukey’s post hoc test for comparison among three or more groups, and two-way ANOVA with Tukey’s post hoc (OriginPro 2022, Origin Lab Corporation) test for comparison between groups on two independent variables. *p*-values of < 0.05 were considered statistically significant. *, *p* < 0.05; **, *p* < 0.01; ***, *p* < 0.001.

Transcriptome. Data analyses on fastq files were conducted with CLC Genomics Workbench (version 20.0.3, QIAGEN, Venlo, The Netherlands). The reads of all probes were adapter-trimmed (Illumina TruSeq) and quality-trimmed (using the default parameters: bases below Q13 were trimmed from the end of the reads, ambiguous nucleotides maximal 2). Mapping was performed against the *C. elegans* (WBcel235.99) (26 March 2020) genome sequence. The GO annotation file wb.gaf (from GeneOntology, 23 March 2020) was used for functional classification. After grouping of samples (six biological replicates each) according to their respective experimental condition, multi-group comparisons were made and statistically determined using the Empirical Analysis of DGE (version 1.1, cutoff = 5). The resulting *p*-values were corrected for multiple testing by FDR and Bonferroni correction. A *p*-value of ≤0.05 was considered significant.

## Data Availability

All data will be made available upon request.

## Supplementary Materials

The following supporting information can be downloaded at: https://www.mdpi.com/article/10.3390/jox14010008/s1, Figure S1: Nano silica induced no branching of PDE dendrites, but dipeptide condensation in intestinal epithelial cells; Figure S2: Patterns of nano silver-induced neurodegeneration during adult life of the nematode *C. elegans*; Figure S3: Transmission electron micrographs (TEMs) of nano silica, BULK silica and nano silver particles; Table S1: Biophysical properties and synthesis of silica and silver particles; Figure S4: Correlation of the phenotype internal hatch with dipeptide condensation and neural beading in serotonergic neurons; Figure S5: Different neurodegenerative fluorescence patterns of the nlp-3p::GFP reporter nematode; Figure S6: Correlation of the internal hatch phenotype with axonal beading of HSN neurons.
