# Supplementary material for: Silica Nanoparticles Disclose a Detailed Neurodegeneration Profile throughout the Life Span of a Model Organism
Source: J Xenobiot. 2024 Jan 12;14(1):135–53. doi: 10.3390/jox14010008 (PMC10801581; doi:10.3390/jox14010008)
Supplement: Supplementary file 1 [file jox-14-00008-s001.zip › jox-2764280-supplementary.pdf]

Supplemental Material

Title

Silica nanoparticles disclose a detailed neurodegeneration profile throughout the life span of a model organism.

Authors

Annette Limke[1], Gereon Poschmann[2], Kai Stühler[3], Patrick Petzsch[4], Thorsten Wachtmeister[4] and Anna von Mikecz[1,*]

Affiliations

[1]IUF – Leibniz Research Institute of Environmental Medicine GmbH, Auf'm Hennekamp 50, 40225 Duesseldorf, Germany

[2]Institute of Molecular Medicine, Proteome Research, Medical Faculty and University Hospital, Heinrich Heine University Düsseldorf, 40225 Düsseldorf, Germany

[3]Molecular Proteomics Laboratory, BMFZ, Heinrich Heine University Düsseldorf, 40225 Düsseldorf, Germany

[4]Biological and Medical Research Center (BMFZ), Medical Faculty, Heinrich-Heine-University, Universitätsstraße 1, 40225 Duesseldorf, Germany

[*]Correspondence: mikecz@tec-source.de; Tel.: +49-221-3389-358

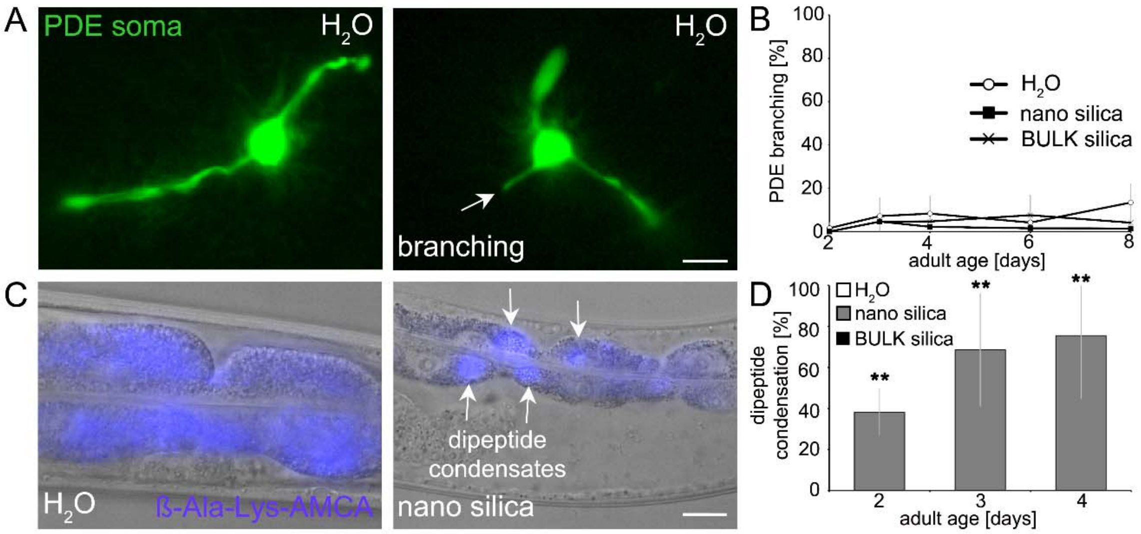

Figure S1. **Nano silica induced no branching of PDE dendrites, but dipeptide condensation in intestinal epithelial cells.** (A) Representative fluorescent micrographs of an 8-day-old, adult reporter worm (dat-1p::GFP) expressing green fluorescent protein (GFP) under control of the dopamine transporter (dat-1) promoter in the soma and dendritic processes of dopaminergic neuron PDE (left). A subset of PDE neurons show outgrowth of additional dendritic processes (right, arrow). (B) Quantification of the PDE branching phenotype in 2- to 8-day-old worms that were mock-exposed (H_2O), or exposed to 200 µg/ mL nano silica or BULK silica. Only low numbers of extra branches were counted in H_2O controls, nano silica-exposed or BULK silica-exposed 2-day- to 8-day-old *C. elegans*. No significant differences were observed between negative controls and particle-treated groups. Values represent means ± SD from 3-6 independent experiments with n=20-27 worms per condition per experiment. Bar, 6 µm. (C) Representative fluorescent micrographs show the localization of fluorescent dipeptide conjugate ß-Ala-Lys-AMCA (blue) in intestinal cells of 4-day-old, adult wild type (N2) *C. elegans*. Nematodes were mock-exposed (H_2O) or exposed to 200 µg/ mL nano silica for 72 hours at 20 °C. ß-Ala-Lays-AMCA staining indicates diffuse peptide distribution in mock-exposed worms (left micrograph) and formation of peptide condensates (arrows) in nano silica-exposed worms (right micrograph). (D) Quantification of dipeptide condensates in 2- to 4-day-old mock-exposed worms or worms exposed to 200 µg/ mL nano or BULK silica. Significant differences were observed between negative controls, BULK silica controls and nano silica-exposed nematodes. Nano silica induced shrinkage of intestinal epithelial cells and redistribution of normally diffusely distributed dipeptides to dipeptide condensates. Bar graphs show mean values ± SD from three independent experiments with n=20-28 per condition per experiment (One-way ANOVA with Tukey's post hoc test). Note that in D values of H_2O and BULK are below 1%. Bar, 10 µm. **, $p<0.01$.

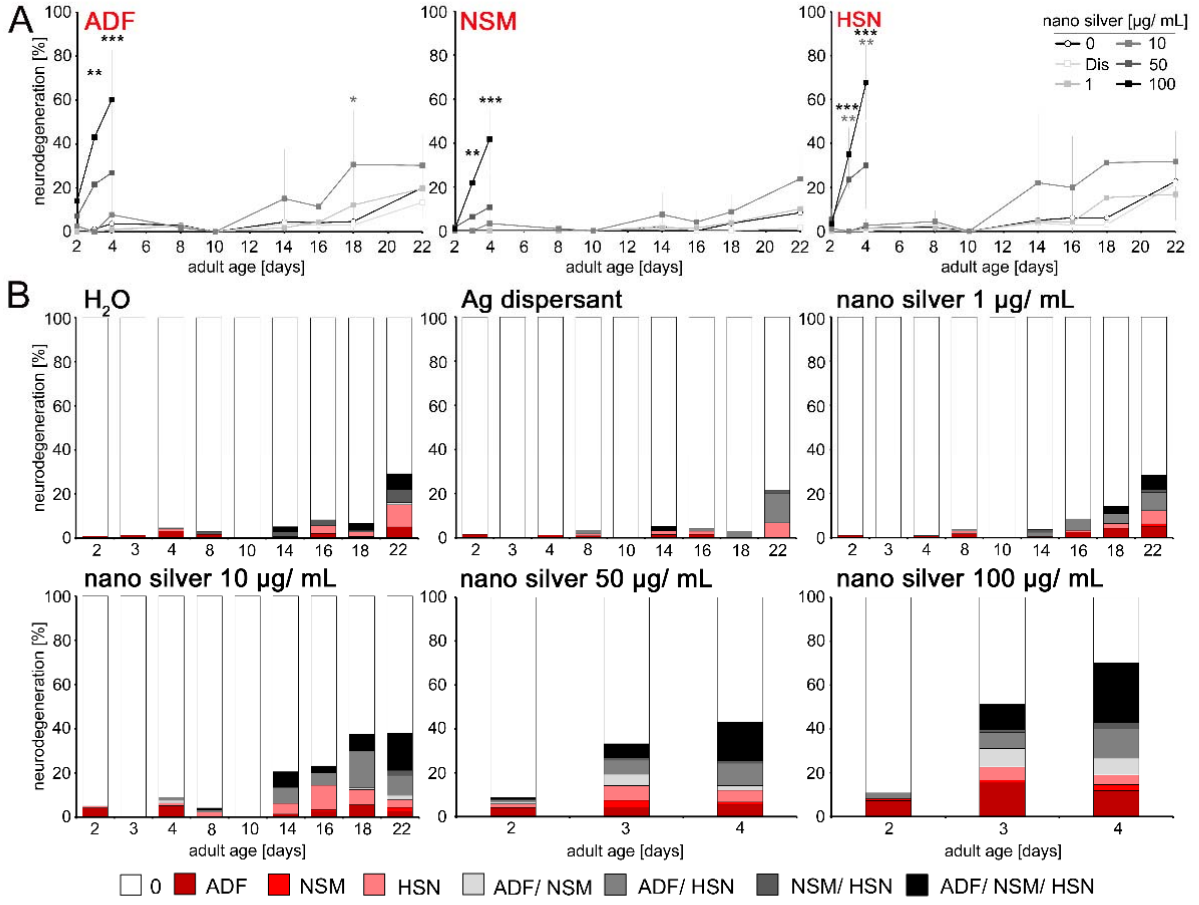

Figure S2. Patterns of nano silver-induced neurodegeneration during adult life of the nematode *C. elegans*.

One-day-old wild-type (N2) nematodes were mock-exposed (H_2O or dispersant) or exposed to increasing concentrations of nano silver. (A) Quantification of neurodegeneration in dendrites of the serotonergic neurons ADF, NSM and HSN in 2- to 22-day-old nematodes. Line graphs represent means ± SD from 4-7 independent experiments with n=7-34 per condition per experiment (One-way ANOVA with Tukey's post hoc test). (B) Quantification of neurodegeneration with respect to specific neurons and exposures. Note, that at concentrations of > 50 µg/ mL neurodegeneration was only observable until day 4 of adulthood due to a high mortality rate of nano silver. At nano silver concentrations between 0 and 10 µg/ mL neurodegeneration was observed predominately in old *C. elegans* affecting single serotonergic neurons or combinations of serotonergic neurons. A significant acceleration of neurodegeneration was observed at 10 µg/ mL nano silver in old (18-day-old) nematodes. Dis, dispersant only; *, $p<0.05$; **, $p<0.01$; ***, $p<0.001$.

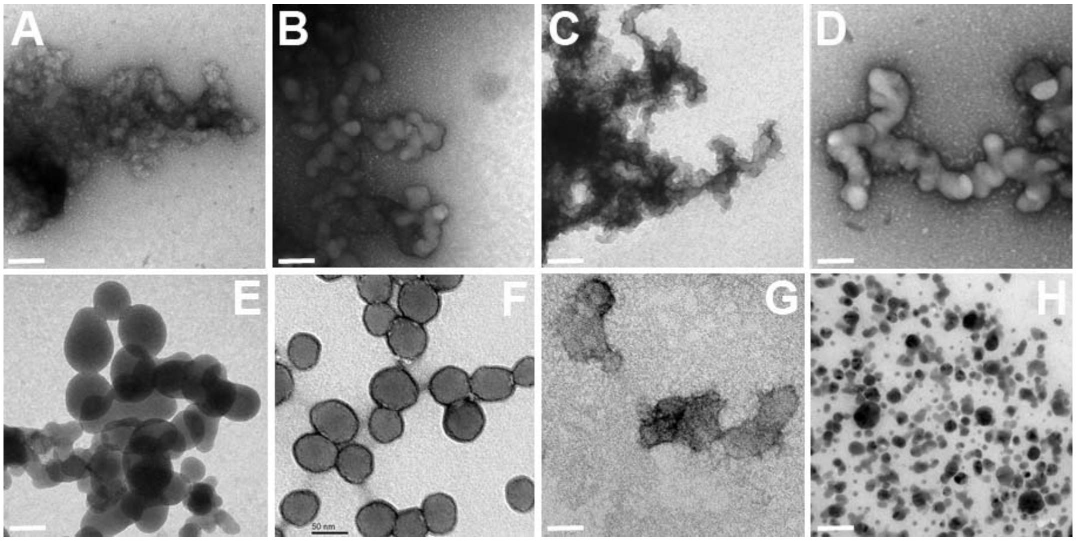

Figure S3 Transmission electron micrographs (TEMs) of nano silica, BULK silica and nano silver particles. (A) 7 nm nano silica, Sigma-Aldrich, (B) 12 nm nano silica, Aerosil 200, Evonic, (C) 14 nm nano silica, Sigma-Aldrich, (D) 20 nm nano silica, Aerosil 90, Evonic, (E) 40 nm nano silica, Aerosil OX50, Evonic, (F) 50 nm nano silica, Kisker, (G) 500-1000 nm BULK silica, Sigma-Aldrich, (H) nano silver, NM300k, European Union Joint Research Center (JRC). Bars, 50 nanometers.

Table S1. Biophysical properties and synthesis of silica and silver particles.

Particles	Diameter, nm	Zeta potential, mV	Synthesis	Source	TEM[1]	Diameter, nm[2]
nano silica	7		HTFH	Sigma-Aldrich	yes	
nano silica	12		HTFH	Aerosil 200, Evonic	yes	
nano silica	14	-9.73	HTFH	Sigma-Aldrich	yes	
nano silica	20	-24.5	HTFH	Aerosil 90, Evonic	yes	
nano silica	40		HTFH	Aerosil OX 50, Evonic	yes	
nano silica	50	-48.7	Stoeber	Kisker Biotech	yes	46 ± 7
BULK silica	200		Stoeber	Kisker Biotech	nd	
BULK silica	500-1000	-11.8	HTFH	Sigma-Aldrich	yes	
nano silver	15	-52.7		EU Joint Research Center	yes	17.24 ± 3.17

[1]TEM, transmission electron microscopy;

[2]The diameter of nanoparticles was determined by dynamic light scattering (DLS).

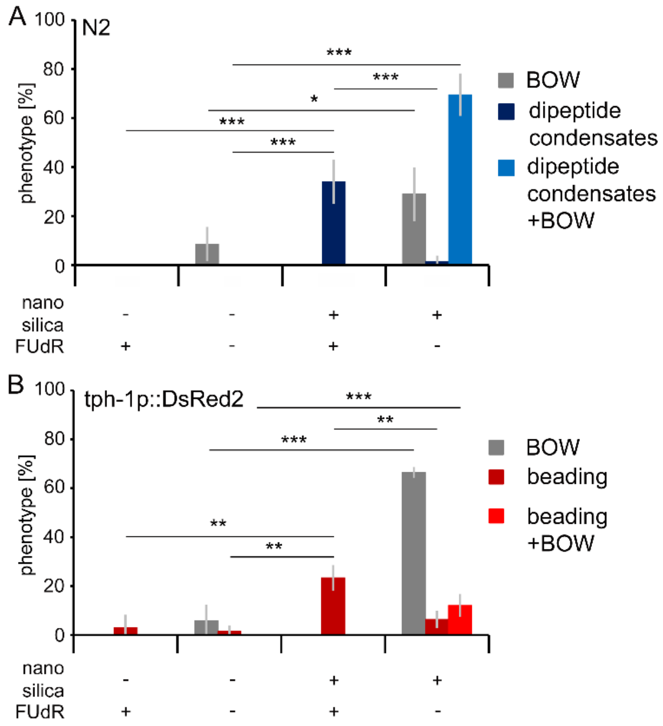

Figure S4. Correlation of the phenotype internal hatch with dipeptide condensation and neural beading in serotonergic neurons. Wild-type (N2) worms (A) or tph-1p::DsRed2 reporter worms (B) were mock-exposed or exposed to 200 µg/ mL nano silica for 26 h with or without FUdR. FUdR treatment suppressed the BOW-phenotype in both strains. (A) Quantification of the BOW-phenotype and dipeptide condensates in 2-day-old, adult wild-type. Nano silica-induced dipeptide condensation occured simultaneously with the BOW-phenotype (without FUdR). Values represent means ± SD from three experiments with n=16-23 for per condition per treatment (Two-way ANOVA with Tukey's post hoc test). (B) Quantification of the BOW-phenotype and neural beading in 2-day-old tph-1p::DsRed2 reporters. Beading in serotonergic neurons occurred independently of the BOW-phenotype. Values represent means ± SD from three experiments with n=16-33 for per condition per treatment (Two-way ANOVA with Tukey's post hoc test). Beading, discontinuous fluorescence pattern of dendrites; BOW, bag-of-worms; FUdR; 5-fluoro-2'-deoxyuridine; *, p<0.05; **, p<0.01; ***, p<0.001.

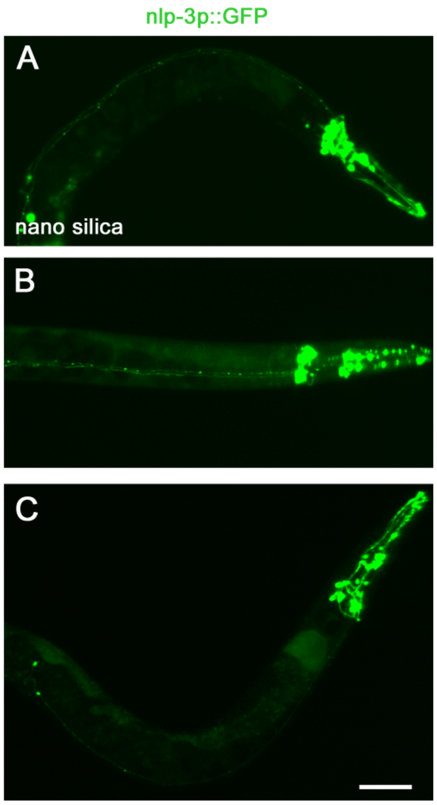

Figure S5. Different neurodegenerative fluorescence patterns of the nlp-3p::GFP reporter nematode. Representative fluorescent micrographs of 8-day-old, adult *C. elegans* expressing the green fluorescent protein (GFP) under the control of the neuropeptide-like protein (nlp-3) promoter in HSN (left) and head neurons (right). Reporter *C. elegans* were treated with 200 μg/ mL nano silica at 20 °C. Nano silica-exposed worms show different fluorescent patterns/ phenotypes: (A) beading (punctea) in the HSN axon, (B) beading (punctea) in the HSN axon and head neurons and (C) loss of neuropeptide expression in the HSN axon. Bar, 50 μm.

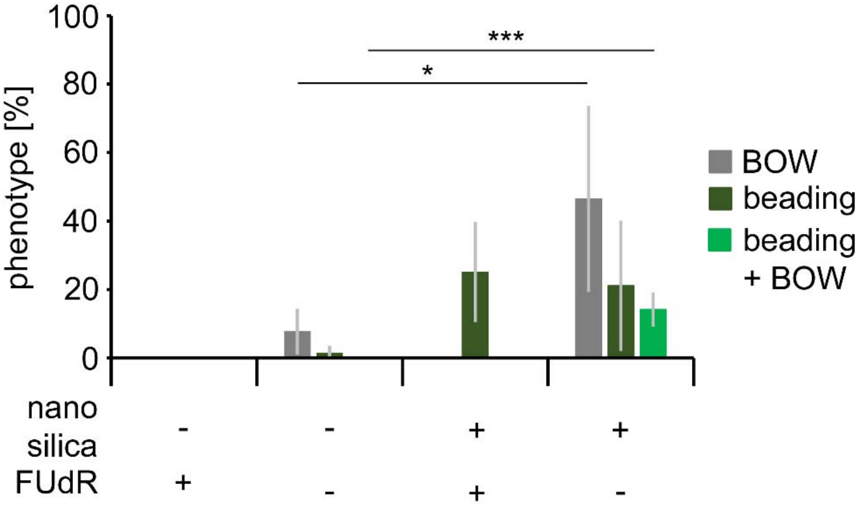

Figure S6. Correlation of the internal hatch phenotype with axonal beading of HSN neurons. Quantification of the BOW-phenotype and axonal beading in 2-day-old, adult nlp-3p::GFP reporter nematodes that were mock-exposed or exposed to 200 µg/ mL nano silica for 26 h with or without FUdR. The FUdR treatment suppressed the BOW-phenotype. Axonal beading in the HSN occurred independently of the BOW-phenotype. Values represent means ± SD from three experiments with n=20-30 for per condition per treatment (Two-way ANOVA with Tukey's post hoc test). BOW, bag-of-worms; FUdR, 5-fluoro-2'-deoxyuridine; *, $p<0.05$; ***, $p<0.001$.
